# Primary Absolute Cardiovascular Disease Risk and Prevention in Relation to Psychological Distress in the Australian Population: A Nationally Representative Cross-Sectional Study

**DOI:** 10.3389/fpubh.2019.00126

**Published:** 2019-05-31

**Authors:** Jennifer Welsh, Rosemary J. Korda, Grace Joshy, Emily Banks

**Affiliations:** ^1^National Centre for Epidemiology and Population Health, Research School of Population Health, Australian National University, Canberra, ACT, Australia; ^2^The Sax Institute, Ultimo, NSW, Australia

**Keywords:** cardiovascular disease, psychological distress, prevention, risk factors, absolute risk

## Abstract

People who experience psychological distress have an elevated risk of incident cardiovascular disease (CVD). However, the extent to which traditional CVD prevention strategies could be used to reduce the CVD burden in this group is unclear because population-level data on CVD risk profiles and appropriate management of risk in relation to distress are currently not available. The aim of this study was to use nationally representative data to quantify variation in CVD risk and appropriate management of risk according to level of psychological distress in the Australian population. Data were from 2,618 participants aged 45–74 years without prior CVD who participated in the 2011-12 Australian Health Survey, a cross-sectional and nationally representative study of Australian adults. Age-and sex-adjusted prevalence of 5-year absolute risk of primary CVD (low <10%, moderate 10–15%, or high >15%), CVD risk factors, blood-pressure, and cholesterol assessments, and appropriate treatment (combined blood pressure- and lipid-lowering medication) if at high primary risk, were estimated. Prevalence ratios (PR) quantified variation in these outcomes in relation to low (Kessler-10 score: 10-<12), mild (12-<16), moderate (16-<22) and high (22–50) psychological distress, after adjusting for sociodemographic characteristics. The prevalence of high absolute risk of primary CVD for low, mild, moderate and high distress was 10.9, 12.3, 11.4, and 18.6%, respectively, and was significantly higher among participants with high compared to low distress (adjusted PR:1.62, 95%CI:1.04–2.52). The prevalence of CVD risk factors was generally higher in those with higher psychological distress. Blood pressure and cholesterol assessments were reported by the majority of participants (>85%) but treatment of high absolute risk was low (<30%), and neither were related to psychological distress. Our findings confirm the importance of recognizing people who experience psychological distress as a high risk group and suggest that at least part of the excess burden of primary CVD events among people with high psychological distress could be reduced with an absolute risk approach to assessment and improved management of high primary CVD risk.

## Introduction

Cardiovascular disease (CVD) is the leading cause of morbidity and mortality internationally (1) and the burden is higher in people with psychological distress. People who experience psychological distress, a term capturing general symptoms of depression and anxiety (2), have a 50% elevated risk of developing CVD (3) and a 2-fold elevated risk of dying from CVD (4) compared to people without distress.

Reasons for the higher burden of CVD among those with distress remain unclear, including the extent to which the association is causal (5). Which strategies are likely to be most effective in reducing the excess CVD incidence among people with higher levels of distress are therefore also unknown (6). Psychological distress is associated with changes to a number of biological systems, including autonomic and immune dysregulation (7). However, whether these changes mediate a causal distress-CVD association, and the extent to which they could be used as therapeutic targets, is yet to be established (6). In contrast, the utility of traditional approaches to CVD prevention are well-documented. Therefore, regardless of whether high psychological distress causes CVD or is a marker of elevated risk for other reasons, if higher incidence among those with distress reflects higher absolute risk of CVD or under treatment of risk, a substantial proportion of the excess cases of CVD could be prevented with traditional approaches to CVD prevention.

Guidelines for CVD prevention in many high income countries, including Australia, the US and the UK, recommend an absolute risk approach to assessment and management (8–10). Absolute CVD risk is the absolute probability of an individual having a CVD event over a given time period, based on a combination of multiple risk factors rather than the presence of single risk factors (8). In Australia, guidelines recommend absolute risk assessment for all people aged 45–74 years using the National Vascular Disease Prevention Alliance (NVDPA) algorithm (8), which uses clinical criteria and the Framingham risk equation (11, 13) to calculate absolute risk of having a CVD event over the next 5-years. Best practice guidelines recommend that people at high absolute risk should be given advice and support to promote positive lifestyle change with combined blood pressure- and lipid-lowering medications, unless contraindicated, or clinically inappropriate (8).

While the prevalence of health conditions known to increase the risk of developing CVD such as hypertension (14), diabetes (15), and chronic kidney disease (16), and behavior-related risk factors (e.g., smoking) (17, 18), are known to be higher among those who experience psychological distress, nationally representative data on CVD risk factor profiles according to level of distress are lacking, and to date no study has quantified whether levels of absolute primary CVD risk vary in relation to level of distress. Also unknown is whether assessment or treatment of absolute primary CVD risk varies in relation to level of psychological distress. People with higher psychological distress are known to utilize health care, including primary health care, more frequently than those with lower levels of distress (19), but whether this supports CVD risk assessment (including blood pressure and cholesterol assessment) and appropriate treatment is unknown.

The primary aim of this paper was to use nationally representative data from Australian adults to quantify risk of a primary CVD event, including 5-year absolute CVD risk and biological and behavioral factors underpinning absolute CVD risk, in relation to level of psychological distress. Our secondary aim was to examine distress-related variation in management of CVD risk, including blood pressure and cholesterol assessment, and treatment of those at high risk.

## Materials and Methods

### Study Design and Sample

Data came from people aged 45–74 years old without prior CVD who took part in the National Health Survey (NHS) and provided biomedical data for the National Health Measures Survey (NHMS) as part of the 2011–12 Australian Health Survey (12). The NHS is a cross-sectional, nationally representative survey of Australian households. Households were selected using a stratified multistage area sample of private dwellings in Australia; one adult (18 years and older) and one child (0–17 years) were randomly selected from each household to participate in the NHS and all participants in the NHS aged 5 years and over were invited to participate in the NHMS (20). Of the 7,171 persons aged 45–74 years who were included in the NHS, 3,255 participated in the NHMS. These participants were included in our study if they did not have prior CVD (self-reported history of ischaemic heart disease, heart failure, other heart disease, cerebrovascular disease, disease of the arteries, arterioles and capillaries, or current and long-term oedema) and had information on all available components of the absolute CVD risk equation.

### Measures

Sociodemographic characteristics and health behaviours were assessed by self-report during home-based interviews. Medications were assessed as part of a medication review. Height and weight, waist circumference and blood pressure were measured directly (21). Participants were classified as obese if their body mass index (BMI) was ≥30 kg/m^2^ and high waist circumference was defined as ≥94 cm for men and ≥80 cm for women. Physical activity, based on duration and intensity of exercise undertaken for fitness, recreation, sport or walking for transport in the last week, was categorized into sedentary or low compared to moderate and high (21). More than two standard drinks per day indicated high alcohol intake (22). Fasting blood and urine samples were collected and assayed to measure HbA1c, fasting plasma glucose, glomerular filtration rate, low density lipoprotein (LDL), high density lipoprotein (HDL), and total cholesterol, and microalbuminuria (23). Participants with HbA1C levels of ≥6.5%, fasting glucose levels of ≥7.0 mmol/L or self-reported diabetes and diabetes medication (insulin and analogs, blood glucose lowering medication, and other drugs in diabetes) were considered to have diabetes. Participants with glomerular filtration level of <45 mL/min/1.73 m^2^ were considered to have moderate to severe chronic kidney disease. LDL cholesterol levels were considered high if >3.5 mmol/L, HDL cholesterol was considered low if <1.0 mmol/L and total cholesterol was high if levels were ≥6.0 mmol/L.

**Figure 1:**
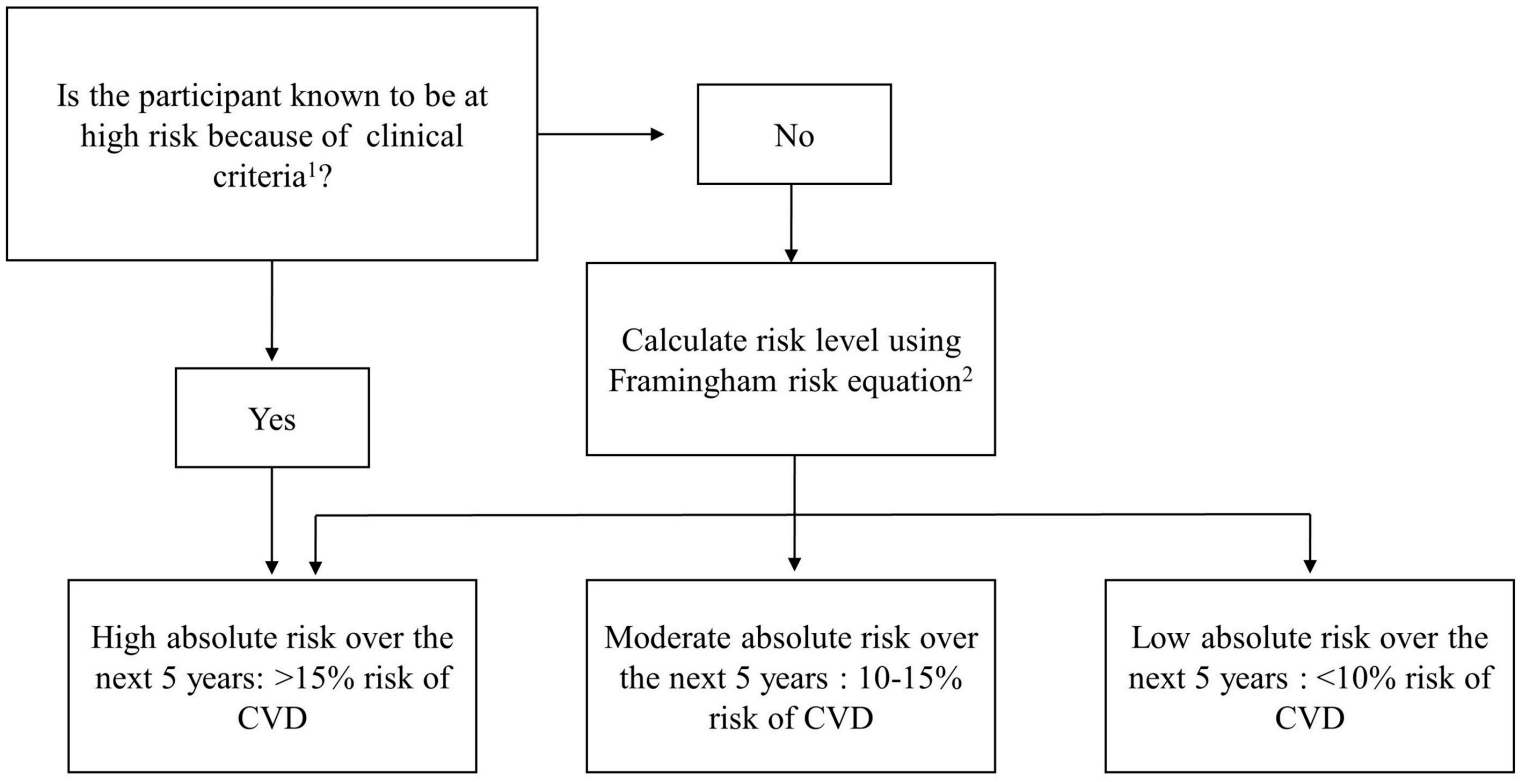
National Vascular Disease Prevention Alliance (NVDPA) absolute CVD risk assessment algorithm. ^1^Participants are automatically determined to be at high risk if they meet one or more of the following clinical criteria: Diabetes and aged over 60 years; diabetes with microalbuminuria; moderate or severe chronic kidney disease; systolic blood pressure ≥180 mmHg or diastolic blood pressure ≥110 mmHg; serum total cholesterol >7.5 mmol/L. ^2^The Framingham risk equation uses information on age, sex, smoking status, total: HDL cholesterol, systolic blood pressure, and diabetes to calculate absolute risk of CVD.

Five-year absolute risk of a primary CVD event was calculated using the NVDPA algorithm. This algorithm combines information on clinical characteristics which automatically confer high risk with the Framingham risk equation to classify participants into low (<10%), moderate (10–15%) and high (>15%) absolute risk of having a CVD event in the next 5-years (Figure 1). Two risk factors usually included in the Framingham risk equation, left ventricular function and familiar hypercholesterolemia, were not available in the study and were not included in the risk equation.

Blood pressure and cholesterol assessments, key components of absolute CVD assessment, were measured by asking participants if they had their blood pressure checked in the previous 2 years and their cholesterol checked in the previous 5 years, based on the minimum recommended national clinical guidelines (24). Participants who reported both checks were considered to have had blood pressure and cholesterol assessments. In line with best practice guidelines, treatment of high absolute risk was measured as simultaneous treatment with blood pressure- and lipid-lowering medication. Medications were classified according to the World Health Organization Anatomical Therapeutic Chemical (ATC) classification system. Blood pressure lowering medication included ATC codes C02, C03, C07 C08, and C09, lipid lowering medication was captured with ATC code C10.

Psychological distress was measured with the Kessler 10 (K10) (25). The K10 measures frequency of non-specific symptoms of psychological distress (such as “depressed,” “nervous,” “worthless”) experienced in the past 4 weeks from 1 “none of the time” to 5 “all of the time.” Scores range from 10 (no distress) to 50 (severe distress), where higher scores indicate a higher likelihood of the presence of a mood/anxiety disorder. Scores were grouped into four categories: low (10-<12), mild (12-<16), moderate (16-<22) and high (22–50) psychological distress.

Sociodemographic characteristics potentially associated with both CVD risk and psychological distress were also measured and grouped into categories. These were: age (measured with 5-year age group), sex (men and women), country of birth (Australian/ New Zealand and other), highest level of education (tertiary, certificate/ diploma/ trade, and high school or less) and region of residence (major city, inner regional and other).

### Statistical Approach

We quantified variation in CVD risk and management of risk, using modified Poisson regression, which is a generalized linear model with a Poisson distribution and a log link function (26). This method produces estimates which can be interpreted as a prevalence ratio (PR), that is, the ratio of those with the outcome to those in the population (27).

**Figure 2:**
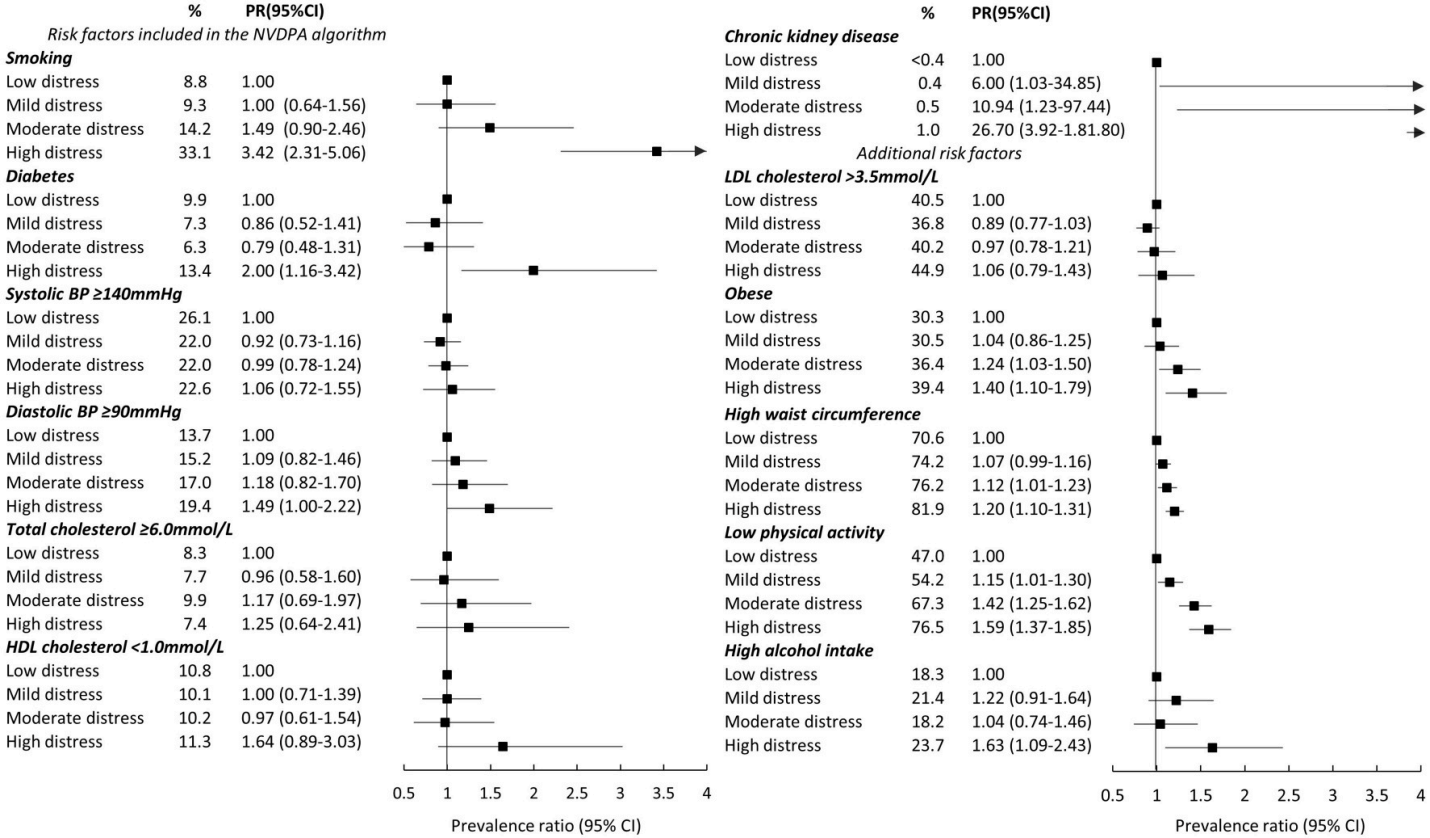
Age- and sex- adjusted prevalence ratios (PR) (and 95% CI) between categories of psychological distress and risk factors for cardiovascular disease. Notes: % are weighted. Obesity is BMI≥30 kg/m^2^. At-risk waist circumference is ≥92 cm for men and ≥80 cm for women. High alcohol intake is more than two standard drinks per day. Standard errors for chronic kidney disease were estimated with robust standard errors because models failed to converge when applying the Jackknife method.

Our analyses were conducted in two stages. In the first stage, we examined associations between levels of distress and CVD risk, including absolute risk of primary CVD and biological and behavioral risk factors underpinning risk. The prevalence of low, moderate, and high absolute risk of primary CVD (based on the NVDPA algorithm) were estimated for the study population, as were age- and sex- adjusted prevalence of each absolute risk category for each category of psychological distress. Prevalence ratios (PR) and 95% confidence intervals (CI), which quantified the association between categories of distress and high primary risk of CVD (5-year absolute risk >15% compared to low or moderate absolute risk ≤ 15%), were also estimated. We then estimated PRs and 95% CIs describing the association between categories of psychological distress and CVD risk factors, including factors in the NVDPA algorithm and additional factors known to increase CVD risk. Each risk factor was dichotomized (see Figure 2 for groupings) with the highest risk category used as the outcome in the analyses.

In the second stage, we examined distress-related variation in appropriate management of risk. Appropriate management requires assessment of absolute CVD risk (requiring at a minimum, blood pressure and cholesterol assessment as well as information on smoking and diabetes status) and, for those found to be at high absolute risk, treatment with combined blood pressure- and lipid-lowering medications. In this stage, we estimated PRs and 95%CIs describing the association between categories of distress and having both blood pressure and cholesterol assessments (compared to not having both assessments), and among those at high primary risk, combined blood pressure- and lipid-lowering medication (compared to neither or only one medication).

All models were adjusted for age and sex. Models predicting high absolute risk of primary CVD, assessment and treatment of high risk were additionally adjusted for highest level of education, country of birth and region of residence to assess the extent to which distress was associated with high absolute risk of primary CVD or management of risk after accounting for basic sociodemographic factors. In all analyses, psychological distress was the exposure variable and low distress was used as the reference category. Where appropriate, tests for linear trend were performed by modeling categories of distress as an ordinal variable. A sensitivity analysis was performed excluding participants with a history of cancer (other than skin cancer) to test whether any observed variation in high absolute risk in relation to categories of distress could be due to a greater proportion of people with high psychological distress having higher absolute risk of CVD because of prior cancer treatment.

After excluding participants with missing data on the available components of the risk equation, there were no missing data on sociodemographic characteristics but there were missing values for LDL cholesterol (*n* missing cases = 595), BMI (*n* = 64), waist circumference (*n* = 47), physical activity (*n* = 3), alcohol intake (*n* = 3), and blood pressure and cholesterol assessments (*n* = 7), which were excluded using list wise deletion. All analyses were weighted to account for the sampling strategy and non-response to the NHMS component of the Survey. Weights, created by the ABS, were based on the inverse probability of the household's and the individual's selection into the study sample. Weights for the NHMS were benchmarked to the estimated resident population living in private dwellings in non-very remote areas of Australia, based on 2006 Census information (28). The delete-a-group Jackknife method (using 60 replicate weights provided by the ABS) was used to estimate standard errors. All analyses were performed using Stata version 15.1 (29) within the ABS DataLab with approval from the ABS and in accordance with the Commonwealth Australian Census and Statistics Act 1905.

Ethics approval for NHMS data collection was provided by the Australian Government Department of Health Human Research Ethics Committee (reference 2/2011). Further ethical approval for this study was obtained from the Australian National University Human Ethics Committee (2010/513) and the NSW Population and Health Services Research Ethics Committee (HREC/10/CIPHS/33; CI NSW Study Reference 2010/05/234).

## Results

Of the 3,255 participants included in the NHMS, 308 (9.5%) were identified as having prior CVD and a further 329 (10.1%) had missing data on a component of the risk equation or the K10 and were excluded from the analysis. This resulted in a final sample of 2,618 participants (1457 women, 1161 men). Of these, 39.2% were classified as having low psychological distress, 33.8% mild, 17.6% moderate and 9.3% high. The sociodemographic characteristics of the sample are presented in Table 1.

**Table 1:** Sociodemographic characteristics of the study population (weighted %).

	**Psychological distress**				**Total**
	**Low *n* = 1,054**	**Mild *n* = 878**	**Moderate *n* = 443**	**High *n* = 243**	***N* = 2,618**
Total	39.2	33.8	17.6	9.3	100
**AGE**					
45–49	21.3	21.5	27.7	26.3	23.0
50–54	16.5	23.6	24.5	25.5	21.1
55–59	16.7	19.6	17.1	21.2	18.2
60–64	18.4	15.6	17.7	14.4	17.0
65–69	15.9	11.4	8.3	8.6	12.4
70–74	11.1	8.4	4.6	4.1	8.4
**SEX**					
Male	52.4	47.3	47.8	28.6	47.6
Female	47.6	52.7	52.2	71.4	52.4
**COUNTRY OF BIRTH**					
Australia/NZ	65.6	73.3	71.2	70.8	69.7
Other	34.4	26.7	28.8	29.2	30.3
**REGION OF RESIDENCE**					
Major city	67.2	67.3	70.9	67.7	68.0
Inner regional	21.5	23.4	22.6	22.9	22.5
Other	11.3	9.2	6.5	9.4	9.6
**EDUCATIONAL QUALIFICATIONS**					
Tertiary	25.8	26.9	23.0	12.1	24.4
Diploma/certificate/trade	38.1	37.1	40.7	36.6	38.1
High school or less	36.1	35.9	36.2	51.3	37.5

### Absolute Risk of Primary CVD

Overall, 79.3% of the study population were at low, 8.8% moderate and 11.9% at high absolute risk of a primary CVD event (Table S1). Approximately two-thirds (68.4%) of the study population classified as being at high absolute risk had clinically determined high risk; however this number was higher (77.7%) among participants with high psychological distress. The majority of all participants who were clinically determined to be at high risk met diabetes criteria: 57.1% had diabetes and were over the age of 60 years and 21.2% had diabetes with microalbuminuria. This was also true among those with high psychological distress (51.3 and 12.9% had diabetes and were over 60 or had diabetes with microalbuminuria, respectively).

Age- and sex-adjusted prevalence of high primary risk of a CVD event within low, mild, moderate and high distress categories were 10.9, 12.3, 11.4, and 18.6%, respectively. Corresponding figures for moderate absolute risk of a CVD event were 7.8, 7.8, 11.3, and 16.6% (Table 2).

**Table 2:** Weighted age- and sex- adjusted prevalence of primary absolute risk of cardiovascular disease in relation to psychological distress.

	**Absolute risk of a primary CVD event**		
	**Low (< 10%)**	**Moderate (10–15%)**	**High (>15%)**
	**% (95%CI)**	**% (95%CI)**	**% (95%CI)**
**PSYCHOLOGICAL DISTRESS**			
Low	81.5 (78.6–84.4)	7.8 (6.1–9.5)	10.9 (8.8–13.0)
Mild	80.5 (76.8–84.3)	7.8 (5.3–10.2)	12.3 (9.4–15.2)
Moderate	77.8 (73.3–82.4)	11.3 (6.9–15.7)	11.4 (7.3–15.6)
High	69.7 (64.3–75.0)	16.6 (8.7–24.5)	18.6 (11.3–25.9)

*Estimates are based on 2,618 respondents. Estimates do not always sum to 100%*.

PRs for high absolute risk of primary CVD after adjusting for age and sex and compared to low distress were 1.13 (95%CI: 0.85–1.51), 1.05 (95%CI: 0.67–1.64), and 1.72 (95%CI: 1.09–2.70) for mild, moderate and high distress, respectively (Table 3). There was no evidence of a linear trend. These PRs did not change materially after additionally taking region of residence, education level and country of birth into account: compared to low, PRs from Model 2 for mild, moderate and high distress were 1.14 (95%CI: 0.86–1.52), 1.04 (95%CI: 0.67–1.62), and 1.62 (95%CI: 1.04–2.52), respectively. The associations between high absolute risk of primary CVD and categories of psychological distress also did not change materially when excluding participants with a prior cancer diagnosis (Table S2).

**Table 3:** Prevalence ratios for high absolute risk of primary CVD in relation to psychological distress.

	**Age-and sex-adjusted prevalence of high primary risk (95%CI)**	**Model 1**	**Model 2**
		**Prevalence ratio (95%CI)**	**Prevalence ratio (95%CI)**
**PSYCHOLOGICAL DISTRESS**			
Low	10.9 (8.8–13.0)	1.00	1.00
Mild	12.3 (9.4–15.2)	1.13 (0.85–1.51)	1.14 (0.86–1.52)
Moderate	11.4 (7.3–15.6)	1.05 (0.67–1.64)	1.04 (0.67–1.62)
High	18.6 (11.3–25.9)	1.72 (1.09–2.70)	1.62 (1.04–2.52)

*Estimates are based on 2,618 respondents. Model 1 is adjusted for age and sex; Model 2 is further adjusted for region of residence, education, country of birth*.

### CVD Risk Factors

Age- and sex-adjusted associations between CVD risk factors and categories of psychological distress are shown in Figure 2 (unadjusted weighted prevalence, Table S3).

A number of risk factors included in the NVDPA algorithm were elevated among people with higher levels of psychological distress. Diabetes was more prevalent among participants reporting high (13.4%) compared to low (9.9%) psychological distress (PR high compared to low: PR = 2.00, 95%CI: 1.16–3.42), as was low HDL cholesterol (PR = 1.64, 95%CI: 0.89–3.03) and high diastolic blood pressure (PR = 1.49, 95%CI: 1.00–2.22). Smoking was particularly common among participants reporting high compared to low distress: one-third (33.1%) of highly distressed participants were current smokers compared to 8.8% with low distress (PR = 3.42, 95%CI: 2.31–5.06). The overall prevalence of chronic kidney disease was low (<1%) but increased with increasing levels of distress (test for trend: *p* < 0.001). There was no association between psychological distress and high systolic blood pressure or high total cholesterol.

Factors not included in the NVDPA algorithm but associated with increased CVD risk were also elevated among those with higher levels of distress. The prevalence of obesity, high waist circumference and low physical activity all increased as psychological distress increased (tests for linear trend: *p* < 0.05) and were relatively common: the prevalence of obesity ranged from 30.3% (low distress) to 39.4% (high distress); between 70.6 and 81.9% had a high waist circumference and the percentage physically inactive ranged from 47.0% (low distress) to 76.5% (high distress). High alcohol intake was more common among those with high compared to low psychological distress (PR = 1.63, 95%CI: 1.09–2.43). There was no association between categories of distress and LDL cholesterol.

### Blood Pressure and Cholesterol Assessments

Most participants (>85%) reported having had both blood pressure and cholesterol assessments within the last 2 and 5 years, respectively (Table 4): 86.7% of participants with low, 85.4% with mild, 86.0% with moderate, and 85.9% with high distress reported blood pressure and cholesterol assessment after adjustment for age and sex. PRs for having both assessments for mild, moderate and high distress were 0.99 (95%CI: 0.92–1.05), 0.99 (95%CI: 0.93–1.06), and 0.99 (95%CI: 0.92–1.07), with no material difference in models further accounting for region of residence, education or country of birth (Model 2).

**Table 4:** Prevalence ratios for blood pressure and cholesterol assessment in relation to psychological distress.

	**Age-and sex-adjusted percentage for both assessments (95%CI)**	**Model 1**	**Model 2**
		**Prevalence ratio (95% CI)**	**Prevalence ratio (95%CI)**
**PSYCHOLOGICAL DISTRESS**			
Low	86.7 (83.1–90.2)	1.00	1.00
Mild	85.4 (81.4–89.4)	0.99 (0.92–1.05)	0.99 (0.93–1.05)
Moderate	86.0 (80.9–91.2)	0.99 (0.93–1.06)	0.99 (0.93–1.06)
High	85.9 (79.9–91.8)	0.99 (0.92–1.07)	1.00 (0.93–1.08)

*Estimates are based on 2,611 respondents who reported information on blood pressure and cholesterol assessments. Model 1 is adjusted for age and sex; Model 2 is further adjusted for region of residence, education, country of birth*.

### Treatment of High Primary Absolute Risk

The overall number of participants at high absolute risk of primary CVD was relatively small (*n* = 349), limiting the precision and certainty of the estimates regarding appropriate treatment of high risk. The age- and sex-adjusted prevalence of recommended treatment within the study population at high absolute risk was low across all categories of distress: 23.7, 24.7, 17.3, and 27.2% of participants at high primary risk with low, mild, moderate, and high psychological distress, respectively, reported taking combined blood pressure- and lipid-lowering medications (Model 2 PRs: 1.00 (95%CI: 0.53–1.90), 0.69 (95%CI: 0.26–1.78), and 1.11 (95%CI: 0.35–3.49) for mild, moderate, and high compared to low psychological distress, respectively) (Table 5).

**Table 5:** Prevalence ratios for combined blood pressure- and lipid- lowering medication among those at high absolute risk of primary CVD in relation to psychological distress.

	**Age-and sex-adjusted percentage taking both medications (95%CI)**	**Model 1**	**Model 2**
		**Prevalence ratio (95% CI)**	**Prevalence ratio (95%CI)**
**PSYCHOLOGICAL DISTRESS**			
Low	23.7 (14.1–33.2)	1.00	1.00
Mild	24.7 (13.1–36.3)	1.04 (0.57–1.91)	1.00 (0.53–1.90)
Moderate	17.3 (0.0–33.9)	0.73 (0.27–2.00)	0.69 (0.26–1.78)
High	27.2 (0.0–55.7)	1.15 (0.35–3.83)	1.11 (0.35–3.49)

*Estimates are based on 349 respondents who were at high absolute risk of primary a CVD event. Model 1 is adjusted for age and sex; Model 2 is further adjusted for region of residence, education, country of birth*.

## Discussion

Almost one-in-five (18.6%) Australians adults aged 45–74 with high psychological distress are at high absolute risk of a primary CVD event in the next 5 years, compared to approximately one-in-ten participants reporting low (10.9%), mild (12.3%), or moderate (11.4%) levels of distress. The prevalence of CVD risk factors was generally higher among those with higher levels of psychological distress. Although we had no information on whether participants had their absolute risk assessed *per se*, a large majority (>85%) of the study population reported having both blood pressure and cholesterol assessments. Yet, around 70% of those at high primary risk were not receiving the guideline-recommended combined blood pressure- and lipid-lowering medication, and this was not associated with psychological distress.

To our knowledge, this is the first study to quantify the burden of absolute CVD risk, and management of risk in relation to psychological distress using a nationally representative sample. Consistent with research suggesting that elevated CVD incidence among people with distress might reflect poorer physical health (30), the majority (77.7%) of highly distressed participants at high primary risk were clinically determined because of chronic health conditions. Physical ill-health and the associated disability are among the strongest predictors of the presence of psychological distress. Diabetes, very high blood pressure, and chronic kidney disease were more common among those with higher levels of distress and more than double the risk of developing CVD (31–33). In addition to these health conditions, one-third of highly distressed participants were current smokers, compared to <15% of participants experiencing low, mild or moderate distress. Smoking is also associated with more than a 2-fold increase in the risk of developing CVD (34) and is heavily weighted in the Framingham risk equation. Other behavioral risk factors not included in the risk equation were also common among people with high distress: 39% were obese, 75% were physically inactive and almost one-quarter (23.7%) drank more than two standard drinks per day. Given that these data were cross-sectional, it is unclear whether these factors are causes or consequences of high psychological distress. Regardless of the direction of the association, they provide an indication of the higher CVD risk observed among those with high compared to lower levels of distress.

The large majority of participants who were at high absolute risk of primary CVD were not receiving the necessary evidence-based treatment, irrespective of level of psychological distress. This was despite most of our study population reporting measurement of blood pressure and cholesterol levels—two assessments required for absolute risk calculation. While we had no data on whether or not absolute risk assessment was conducted, previous studies from Australia, Britain and Germany indicate that fewer than half of general practitioners routinely conduct absolute CVD risk assessment (35–37) and continue to favor management of individual risk factors rather than absolute risk (38). Although our sample size limited the precision of our results regarding differences in treatment between distress categories, previous US based research has demonstrated that use of blood pressure- and lipid-lowering medication does not vary according to level of distress (39). Treatment gaps among those at high absolute risk have been previously reported in Australia (40) and highlight the ongoing opportunity for CVD prevention.

As efforts continue in high income countries to increase population-based screening for CVD and reduce the overall burden of the disease, our findings confirm the importance of recognizing people with psychological distress as a high risk group. They also indicate that traditional CVD prevention strategies are likely to be effective in reducing the disproportionately high rates of primary CVD events observed among people with high psychological distress. CVD is highly preventable by reducing behavioral risk factors (41, 42) and lowering blood pressure and cholesterol levels with medication with greater absolute benefit for those at high absolute risk (32, 43). Our study demonstrated that a higher proportion of people with high compared to lower levels of psychological distress were at high absolute risk of primary CVD and that appropriate treatment of high absolute risk was low overall. This suggests that irrespective of whether a causal association exists between distress and the development of CVD, an absolute risk approach to assessment and management will reduce CVD events, with disproportionate benefits for people with high distress. An absolute risk approach includes support to reduce behavioral risk factors and treatment of high risk with combined blood pressure- and lipid-lowering medication. Given that this study did not investigate direct biological pathways between distress and CVD, we cannot rule out that there would be additional benefits of primary prevention strategies tailored for this group, such as medication to reduce distress or stress reduction techniques.

In this population-based study of Australian adults, the majority of the data were collected directly or through biological measures. However, information on some behavioral risk factors, and blood pressure and cholesterol assessments was collected through self-report. The overall response rate for the NHS was 85% and was considerably lower (37%) for the NHMS (44). Although population weights were applied to correct for this, generalizations to the total population should be made with this in mind. Our estimates of medication use among those at high primary risk were based on small numbers of participants (*n* = 349), resulting in a higher degree of uncertainty in the estimates. Our study is also likely to have underestimated risk because some factors used to calculate absolute risk—left ventricular function and familial hypercholesterolemia—were not available. Future research is needed to provide large scale data and more certainty in the estimates regarding variation in treatment levels in relation to levels of psychological distress.

## Conclusion

A greater proportion of people who experience high compared to lower levels of psychological distress are at high primary risk of a CVD event and most were not receiving guideline-recommended treatment. Our findings confirm the importance of recognizing people who experience psychological distress as a high risk group and suggest that the excess burden of CVD events among people with high psychological distress could be reduced with an absolute risk approach to assessment and improved management of high primary CVD risk.

## Data Availability

The dataset analyzed for this study is available from the Australian Bureau of Statistics. Information regarding access is available here: https://abs.gov.au/websitedbs/D3310114.nsf/home/About+CURF+Microdata.

## Ethics Statement

Ethics approval for NHMS data collection was provided by the Australian Government Department of Health Human Research Ethics Committee (reference 2/2011). Further ethical approval for this study was obtained from the Australian National University Human Ethics Committee (2010/513) and the NSW Population and Health Services Research Ethics Committee (HREC/10/CIPHS/33; CI NSW Study Reference 2010/05/234).

## Author Contributions

JW conducted the analyses and drafted the manuscript. EB, RK, and JW designed the analyses. GJ provided statistical support. All authors interpreted the data and critically revised and approved the final manuscript.

### Conflict of Interest Statement

The authors declare that the research was conducted in the absence of any commercial or financial relationships that could be construed as a potential conflict of interest.

## Abbreviations

CVD: cardiovascular disease
PR: Prevalence ratio
CI: confidence intervals
NVDPA: National Vascular Disease Prevention Alliance
NHS: National Health Survey
NHMS: National Health Measures Survey
BMI: body mass index
HbA1c: Hemoglobin A1c
K10: Kessler 10
LDL: low-density lipoproteins
HDL: High-density lipoproteins
ATC: Anatomical Therapeutic Chemical.
