# Supplementary material for: Primary Absolute Cardiovascular Disease Risk and Prevention in Relation to Psychological Distress in the Australian Population: A Nationally Representative Cross-Sectional Study
Source: Front Public Health. 2019 May 31;7:126. doi: 10.3389/fpubh.2019.00126 (PMC6554659; doi:10.3389/fpubh.2019.00126)
Supplement: Supplementary file 1 [file Data_Sheet_1.docx]

Primary absolute cardiovascular disease risk and prevention in relation to psychological distress in the Australian population: a nationally representative cross-sectional study

Supplementary Material

# Supplementary Tables

1.1 Table S1. Weighted population prevalence of each absolute risk of cardiovascular disease category in relation to psychological distress.

|  | Absolute Risk of Primary CVD | | | | |
| --- | --- | --- | --- | --- | --- |
|  | Low | Moderate | High | | |
|  |  |  | Clinically determined | FRE determined | Total High |
|  | % (95%CI) | % (95%CI) | % (95%CI) | % (95%CI) | % (95%CI) |
| Psychological Distress | |  |  |  |  |
| Low | 77.6 (74.6-80.6) | 9.4 (7.4-11.4) | 8.7 (6.5-10.9) | 4.3 (2.7-6.0) | 13.0 (10.4-15.5) |
| Mild | 80.9 (76.8-85.0) | 7.3 (4.8-9.8) | 8.0 (5.4-10.6) | 3.8 (2.3-6.4) | 11.8 (8.8-14.7) |
| Moderate | 81.0 (75.8-86.1) | 9.7 (5.8-13.6) | 6.3 (3.9-8.6) | 3.1 (0.9-5.2) | 9.3 (5.8-12.8) |
| High | 77.1 (70.0-84.2) | 10.4 (4.6-16.1) | 9.9 (4.5-15.0) | 2.8 (0.7-4.9) | 12.5 (6.8-18.3) |
| Total | 79.3 (77.6-80.9) | 8.8 (7.4-10.3) | 8.1 (6.7-9.5) | 3.6 (2.8-4.7) | 11.9 (10.4-13.4) |

Notes: Estimates are based on 2618 respondents. FRE Framingham risk equation.

1.2 Table S2. Prevalence ratios for high absolute risk of primary CVD in relation to psychological distress excluding respondents with a prior cancer diagnosis.

|  | Age-and sex-adjusted prevalence high primary risk (95%CI) | Model 1 | Model 2 |
| --- | --- | --- | --- |
|  |  | Prevalence ratio (95%CI) | Prevalence ratio (95%CI) |
| Psychological distress | |  |  |
| Low | 10.6 (8.3-12.9) | 1.00 | 1.00 |
| Mild | 11.8 (8.8-14.9) | 1.11 (0.81-1.52) | 1.10 (0.80-1.51) |
| Moderate | 10.5 (6.4-14.5) | 0.98 (0.60-1.61) | 0.97 (0.60-1.56) |
| High | 17.5 (10.3-24.7) | 1.64 (1.04-2.60) | 1.56 (0.99-2.47) |

Notes: Estimates are based on 2404 respondents. Model 1 is adjusted for age and sex; Model 2 is further adjusted for region of residence, education, country of birth.

1.3 Table S3. Weighted population percentage reporting each CVD risk factor in relation to psychological distress

|  | Psychological Distress | | | | Total |
| --- | --- | --- | --- | --- | --- |
|  | Low | Mild | Moderate | High |  |
| BMI |  |  |  |  |  |
| Normal | 28.7 | 28.7 | 24.8 | 31.8 | 28.3 |
| Overweight | 41.0 | 40.9 | 38.8 | 28.8 | 39.4 |
| Obese | 30.3 | 30.5 | 36.4 | 39.4 | 32.3 |
| Waist circumference |  |  |  |  |  |
| Not at-risk | 29.4 | 25.8 | 23.8 | 18.1 | 26.2 |
| At-risk | 70.6 | 74.2 | 76.2 | 81.9 | 73.8 |
| Physical activity |  |  |  |  |  |
| High | 15.8 | 10.0 | 8.9 | 2.6 | 11.4 |
| Moderate | 37.2 | 35.8 | 23.7 | 21.0 | 32.8 |
| Low | 30.2 | 41.4 | 45.0 | 47.3 | 38.2 |
| Sedentary | 16.8 | 12.8 | 22.3 | 29.2 | 17.6 |
| Alcohol intake per day |  |  |  |  |  |
| ≤2 standard drinks | 81.7 | 78.6 | 81.8 | 76.3 | 80.2 |
| >2 standard drinks | 18.3 | 21.4 | 18.2 | 23.7 | 19.8 |
| Smoking status |  |  |  |  |  |
| Current | 8.8 | 9.3 | 14.2 | 33.1 | 12.2 |
| Former | 39.2 | 39.3 | 35.4 | 26.4 | 37.4 |
| Never | 52.0 | 51.4 | 50.4 | 40.5 | 50.5 |
| Systolic blood pressure (mmHg) |  |  |  |  |  |
| <120 | 37.3 | 37.1 | 40.4 | 34.7 | 37.5 |
| 120-139 | 36.6 | 40.8 | 37.6 | 42.7 | 38.8 |
| 140-179 | 25.5 | 21.2 | 20.9 | 20.9 | 22.8 |
| ≥180 | 0.6 | 0.9 | 1.1 | 1.6 | 0.9 |
| Diastolic blood pressure (mmHg) |  |  |  |  |  |
| <90 | 86.3 | 84.8 | 83.0 | 80.6 | 84.7 |
| ≥90 | 13.7 | 15.2 | 17.0 | 19.4 | 15.3 |
| LDL Cholesterol (mmol/L) |  |  |  |  |  |
| <2.0 | 4.6 | 4.0 | 3.7 | 4.4 | 4.2 |
| 2.0-3.5 | 54.9 | 59.2 | 56.1 | 50.7 | 56.1 |
| >3.5 | 40.5 | 36.8 | 40.2 | 44.9 | 39.6 |
| HDL Cholesterol (mmol/L) |  |  |  |  |  |
| ≥1.0 | 89.2 | 89.9 | 89.8 | 88.7 | 89.5 |
| <1.0 | 10.8 | 10.1 | 10.2 | 11.3 | 10.5 |
| Total Cholesterol (mmol/L) |  |  |  |  |  |
| <4.0 | 65.6 | 68.3 | 64.4 | 67.4 | 66.5 |
| 4.0-<6.0 | 26.1 | 24.1 | 25.7 | 25.2 | 25.3 |
| ≥6.0 | 8.3 | 7.7 | 9.9 | 7.4 | 8.3 |
| Diabetes | 9.9 | 7.3 | 6.3 | 13.4 | 8.7 |
| Diabetes with microalbuminuria | 1.5 | 2.3 | 1.3 | 1.3 | 1.7 |
| Chronic kidney disease | n/a | 0.4 | 0.5 | 1.0 | n/a |

Notes: Obesity is BMI≥30kg/m^2^. At-risk waist circumference is ≥92cm for men and ≥80cm for women. High alcohol intake is more than two standard drinks per day. % of missing cases: obesity 2.4%; at-risk waist circumference 1.8%; physical inactivity 0.1%; alcohol intake 0.1%. LDL cholesterol 22.7%. N/a refers to percentage which has been suppressed due to small cell size.
